# Fabrication of modified hydrogenated castor oil/GPTMS-ZnO composites and effect on UV resistance of leather

**DOI:** 10.1038/s41598-017-03879-3

**Published:** 2017-06-16

**Authors:** Jianzhong Ma, Limin Duan, Juan Lu, Bin Lyu, Dangge Gao, Xionghu Wu

**Affiliations:** 1 College of Resources and Environment, Shaanxi University of Science and Technology, Xi’an, 710021 PR China; 2 Shaanxi Research Institute of Agricultural Products Processing Technology, Xi’an, 710021 PR China; 3 China Leather and Footwear Industry Research Institute, Beijing, 100016 PR China

## Abstract

Leather products are made from the natural skin collagen fibers. It is vulnerable to the environmental factor such as solar ultraviolet irradiation in the using process. Therefore anti-UV performance is a very important quality, particularly for chrome-free leather. ZnO is a well-known UV absorber commonly used in the cosmetic industry. We have investigated its potential to increase the anti-UV performance of chrome-free leather. Modified hydrogenated castor oil/GPTMS-ZnO (MHCO/ GPTMS-ZnO) composites were prepared using spherical ZnO nanoparticles, hydrogenated castor oil, maleic anhydride and sodium bisulfite. MHCO/GPTMS-ZnO composites have better anti-UV ability and stability. MHCO/GPTMS-ZnO composites were applied to the leather processing. The treated samples were exposed to artificial sunlight. Anti-yellowing tests showed that MHCO/GPTMS-ZnO composites significantly improved anti-UV performance of leather.

## Introduction

Researches into the influence of UV radiation on leather collagen have been carried out for a long period of time^1–5^. Studies have found that UV irradiation caused changes in collagen structure, mainly reflected in the reduction of hydrogen bond in the intra and inter collagen molecules, fracture of collagen molecules backbone such as C-N, C=C and C=O bond and the decreasing of COOH, C=O and -COO side chain groups on the peptide bond. In addition, the main beta bond cleavage chain formed a large number of free radicals which accelerated the degradation of collagen molecules and change of collagen structure, thus transforming the triple helix structure of collagen molecules into random coil structure^6^. Currently, researchers are focusing on the impact of ultraviolet radiation on collagen structure, but few reports are available on the study of anti-UV performance of leather chemicals^7^. There are many challenges of doing further work in this area.

Fatliquoring agent is leather chemicals, which can lubricate the leather fibers to endow leather with the necessary softness and flexibility. Traditional fatliquoring agent tends to select crude oil containing a lot of unsaturated double bonds, such as castor oil to synthesize fatliquoring agent to increase the bonding strength of collagen fibers and fatliquoring agent. However, the disadvantage of this oil for fatliquoring application is its unsaturated double bonds, susceptible to oxidation under UV irradiation, which may cause the white or light colored leather to turn yellowing. Hydrogenated castor oil is a valuable derivative from castor oil, which is obtained by catalytic hydrogenation of castor oil. It greatly reduces the unsaturated double bond in castor oil molecules, so that the possibility of double bond inducing leather yellowing diminishes.

ZnO is a multifunctional material, which has attracted extensive research interest for its potential applications^8^. ZnO has been used for the preparation of antibacterial leather and fabric coatings^9, 10^, to construct a super-hydrophobic surface of the fabric^11^ and build the structure of the photoelectric materials^12^. ZnO is a broad-spectrum UV absorber which effectively attenuates UV radiation in both the UVA (320–400 nm) and UVB range (290–320 nm)^13^. Adding ZnO in leather chemicals preparation can improve the anti-UV performance of leather chemicals.

Herein, we present an easy strategy to fabricate the composites based on hydrogenated castor oil and spherical ZnO nanoparticles. This strategy briefly comprises the modification of spherical ZnO nanoparticles with 3-Glycidoxy propyl trimethoxy silane (GTPMS-ZnO), preparation of modified hydrogenated castor oil/GPTMS-ZnO (MHCO/GPTMS-ZnO) composites and its application in leather processing. The anti-UV ability of MHCO/GPTMS-ZnO composites has been investigated, anti-yellowing performance of leather fibers treated with MHCO/GPTMS-ZnO composites has been observed, and then the softness and mechanical properties of leather have been tested. This study will also provide a necessary guidance for the research and development of anti-yellowing leather chemicals.

## Results and Discussion

### Formation principle of MHCO/GPTMS-ZnO composites

The formation of MHCO/GPTMS-ZnO composites via *in situ* method consists of three processes. The first process is the formation of succinic acid esterifying HCO/GPTMS-ZnO (SHCO/GPTMS-ZnO). At first, HCO and GPTMS-ZnO (The amount of GPTMS-ZnO used in this system is 4% of HCO) are mixed under heating and stirring. When maleic anhydride is added into the above system, the chemical reaction will be caused. It will react with hydroxyl groups in the HCO molecules, forming succinic acid esterifying HCO (SHCO). As a consequence, the novel ester bond, double bond and carboxyl groups are successfully transformed into HCO molecules.

The second process is the formation of succinic acid esterification-sulfonation HCO/GPTMS-ZnO (SSHCO/GPTMS-ZnO). In this process, NH_3_·H_2_O, NaHSO_3_ and water are added into the reaction system. NH_3_·H_2_O in water can be decomposed into NH_4_^+^ and OH^−^, which may adjust pH of the reaction system and promote the sulfonation reaction of NaHSO_3_ and SHCO. As a sulfonation reaction product, SSHCO has a characteristic of surfactant agents due to the introduction of hydrophilic sulfonic acid groups. Meanwhile, a large amount of water is added, causing oil in water system to be formed. SSHCO as a dispersing agent or surfactant agent, which contains massive sulfonic acid groups, is enwrapped on the surface of GPTMS-ZnO and HCO.

**Figure 1:**
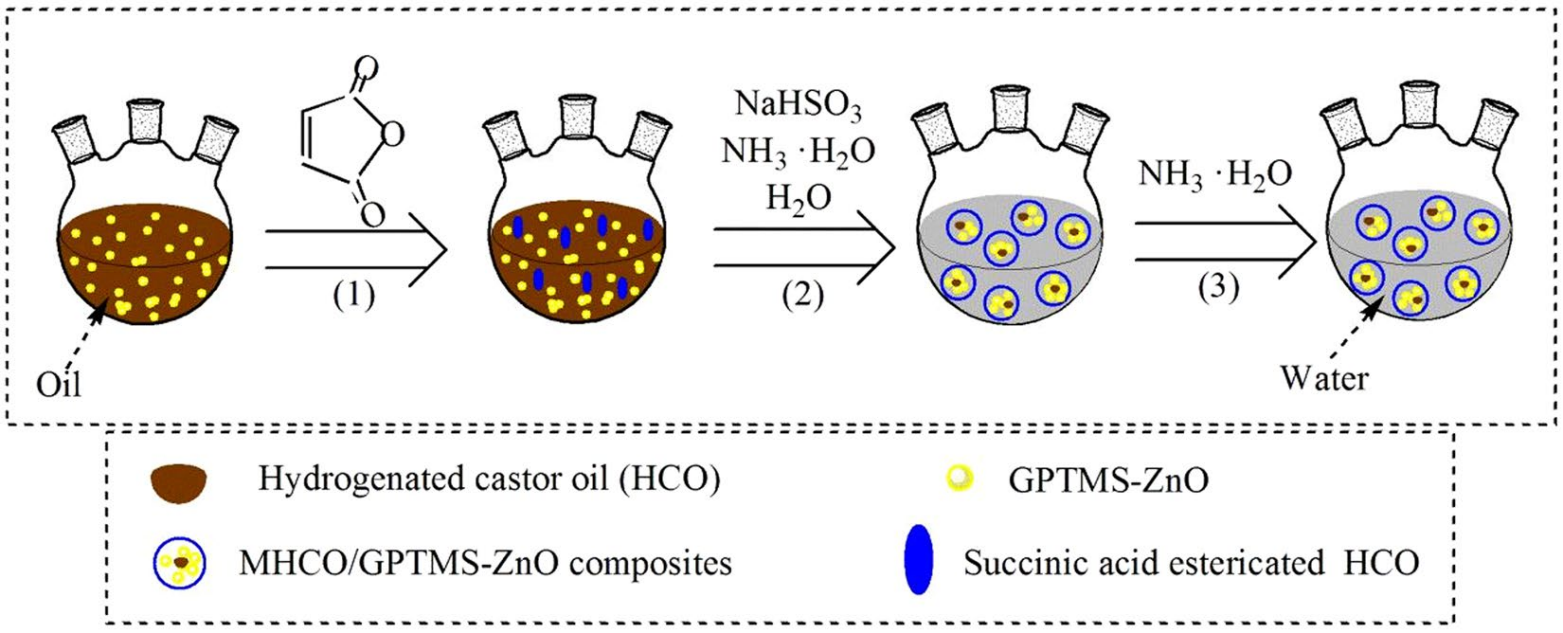
Schematic illustration of the formation of MHCO/GPTMS-ZnO composites.

**Figure 2:**
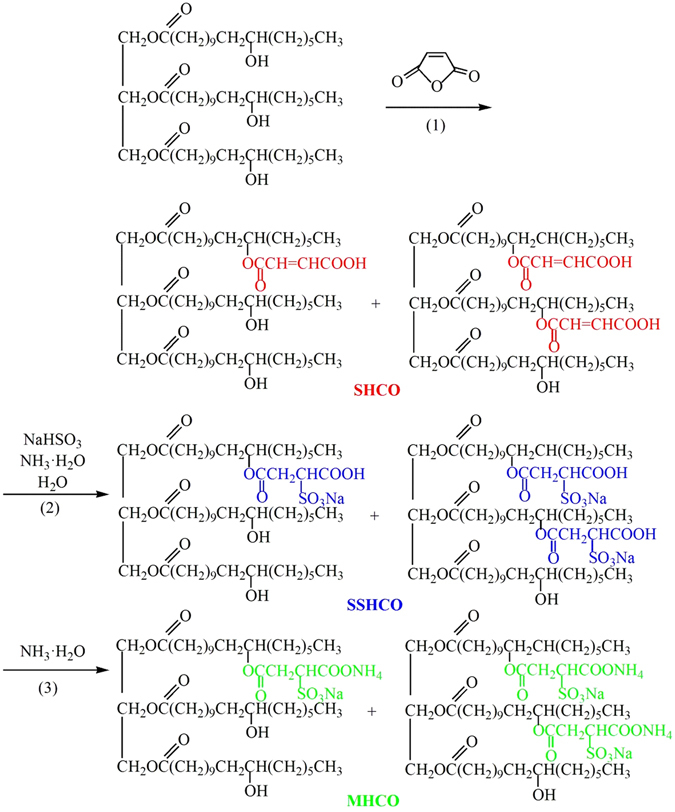
Reaction formula of the preparation of MHCO/GPTMS-ZnO composites.

The last process is the formation of MHCO/GPTMS-ZnO composites. The purpose of this process is to further increase the hydrophilicity of SSHCO to maintain adequate colloidal stability of MHCO/GPTMS-ZnO composites by transforming carboxyl groups into ammonium carboxylate groups. NH_3_·H_2_O is added into the reaction system and a large amount of NH_4_^+^ will make carboxyl groups transform into ammonium carboxylate groups. When SSHCO forms micelles, GPTMS-ZnO and part of HCO will spread to the micelles through aqueous phase and form MHCO/GPTMS-ZnO composites. Figure 1 illustrates the whole formation process of MHCO/GPTMS-ZnO composites and Fig. 2 shows the reaction formula of the preparation of MHCO/GPTMS-ZnO composites.

### Chemical structure and morphology of MHCO/GPTMS-ZnO composites

**Figure 3:**
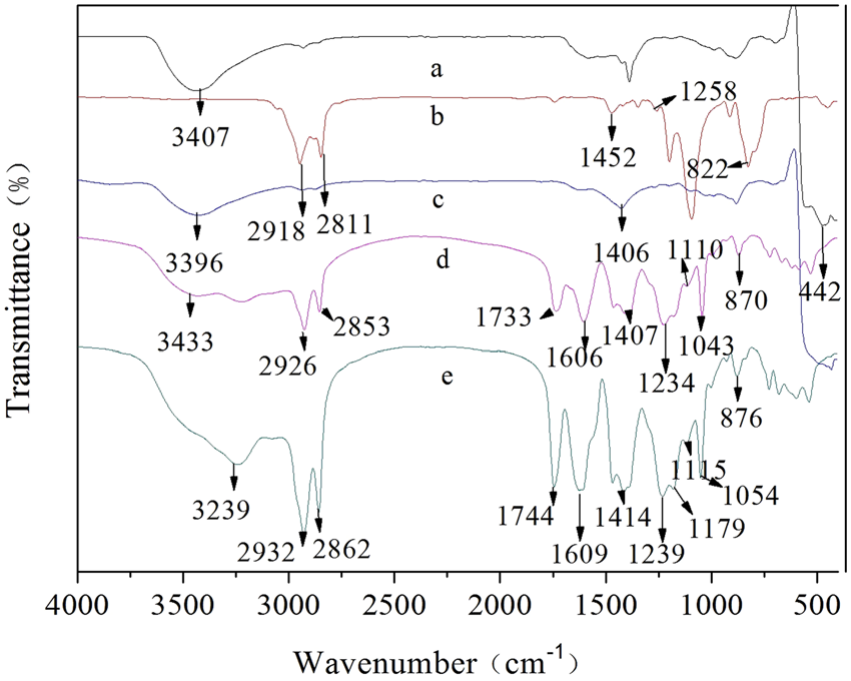
FTIR spectra of (**a**) ZnO, (**b**) GPTMS, (**c**) GPTMS-ZnO, (**d**) MHCO and (**e**) MHCO /GPTMS-ZnO.

**Figure 4:**
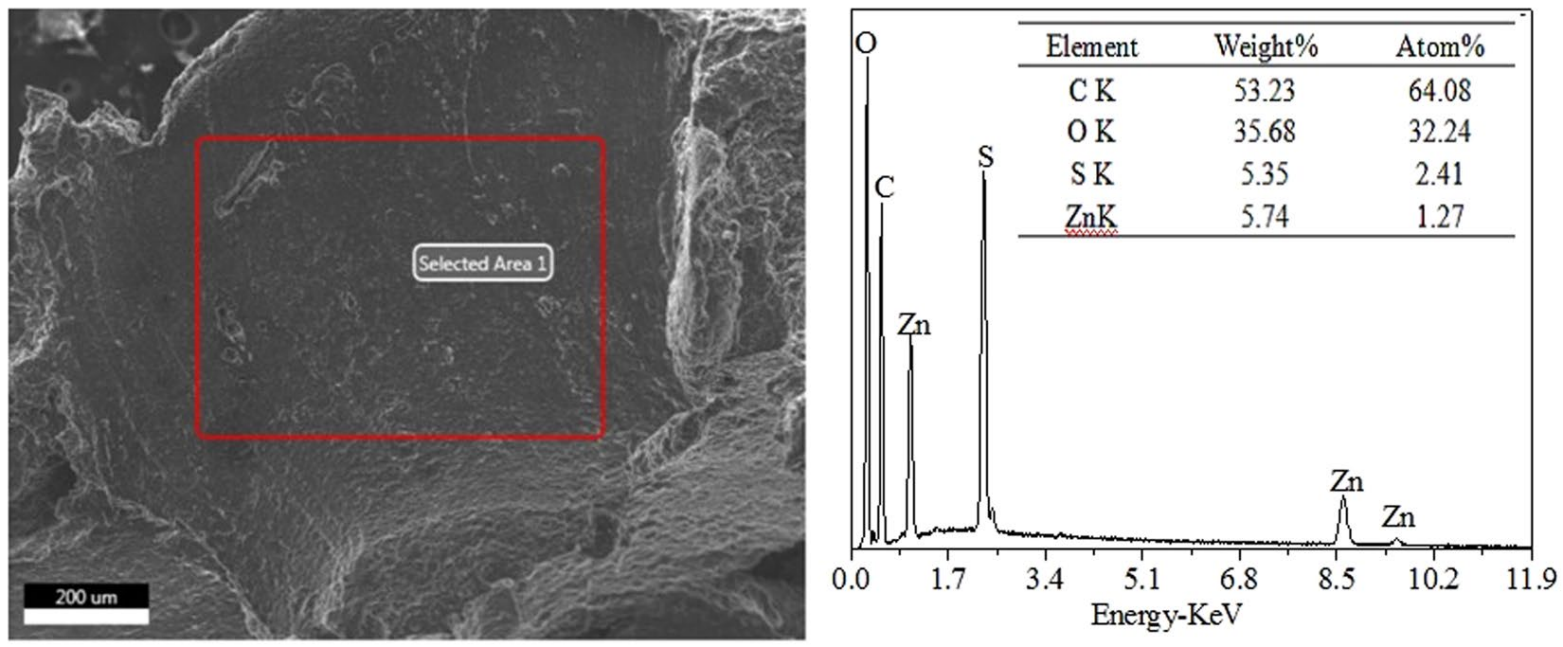
SEM-EDS of MHCO/GPTMS-ZnO composites.

The FT-IR spectra of (a) bare ZnO, (b) GPTMS, (c) GPTMS-modified ZnO, (d) MHCO and (e) MHCO/GPTMS-ZnO composites are shown in Fig. 3. In the spectrum of bare ZnO (Fig. 3a), the broad absorption peak at about 3407 cm^−1^ is hydroxyl group and the peak at 442 cm^−1^ corresponds to the characteristic absorption peak of the Zn-O bond^14^. In the spectrum of GPTMS (Fig. 3b), the absorption bands occurring at 2918 and 2811 cm^−1^ are ascribed to the stretching vibration of -CH_2_, and the peaks at 1452, 1258 and 822 cm^−1^ belong to -CH_2_, epoxy group^15^ and-Si-C^8^, respectively. In the spectrum of GPTMS-modified ZnO (Fig. 3c), the peaks assigned to GPTMS at 1406 and 1272 cm^−1^, and the peak assigned to ZnO broadened and shifted to 3396 cm^−1^, which gave evidence of the reaction of ZnO hydroxyl groups with GPTMS. Figure 3d represents the spectrum of MHCO. The spectrum exhibits absorptions at 2926 and 2853 cm^−1^(-CH_3_, -CH_2_ stretching vibration), 1733cm^−1^ (-C=O stretching vibration), 1234 cm^−1^ (-C-O-C- stretching vibration), 1606cm^−1^ (-COOH stretching vibration), 3433 and 1407 cm^−1^ (-NH_2_ stretching), 1110 and 1043 cm^−1^ (-SO_3_H stretching vibration). It can be concluded that the -COONH_4_ and -SO_3_H have been introduced onto the composites surface. Figure 3e is FT-IR spectrum of MHCO/GPTMS-ZnO composites, which represents -CH_3_ and -CH_2_ stretching vibration absorptions bond (3239 and 2862 cm^−1^), -C=O (1744 cm^−1^), -C-O-C- (1239 cm^−1^), -COOH (1609 cm^−1^), -NH_2_ (3239 cm^−1^), -SO_3_H (1115 and 1054 cm^−1^). The FT-IR spectrum of MHCO/GPTMS-ZnO composites was noteworthy that the characteristic absorption band for C=C at about 1650 cm^−1^ has not been observed, which indicates that MHCO has a high saturation. Figure 3e represents the spectrum of MHCO/GPTMS-ZnO composites, which corresponds with spectrum of MHCO. However, the peaks assigned to ZnO have not been found in the spectrum of MHCO/GPTMS-ZnO composites. It may be because the amount of ZnO in MHCO/GPTMS-ZnO composites is low. In order to prove the existence of ZnO in the MHCO/GPTMS-ZnO composites, MHCO/GPTMS-ZnO composites are characterized by SEM-EDS in Fig. 4. Results show that C atom fraction, O atom fraction, S atom fraction and Zn atom fraction are 64.08%, 32.24%, 2.41% and 1.27%, respectively. C and O elements are from hydrogenated castor oil and S element is from sodium bisulfite. Zn element is from GPTMS-ZnO. Therefore analysis result proves that ZnO exists in MHCO/GPTMS-ZnO composites.

**Figure 5:**
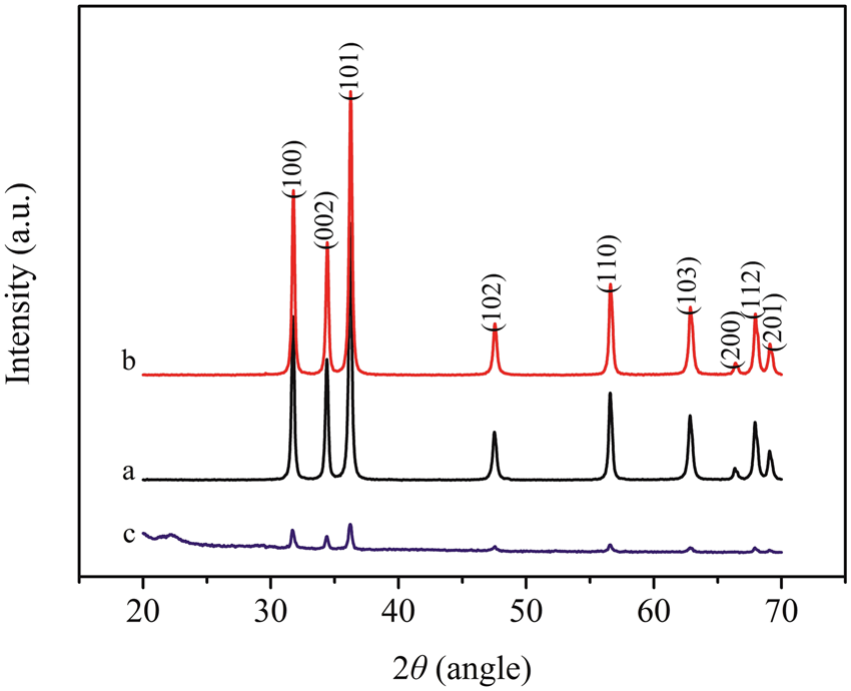
XRD patterns of (**a**) bare ZnO, (**b**) GPTMS-ZnO and (**c**) MHCO/GPTMS-ZnO.

The GPTMS-ZnO and bare ZnO are used as blank control to prove the existence of GPTMS-ZnO in MHCO/GPTMS-ZnO composites. Figure 5 shows the XRD patterns of (a) bare ZnO, (b) GPTMS-ZnO and (c) MHCO/GPTMS-ZnO. From Fig. 5a, a series of characteristic peaks at 2*θ* = 31.9° (100), 33.3° (002), 36.4° (101), 47.7° (102) and 56.7° (110) are observed and they are in accordance with the zincite phase of ZnO. Figure 5b illustrates that after modification, the characteristics peaks position of ZnO remained invariant and is in accordance with the zincite phase of ZnO. This indicates that the grafted silane group doesn’t influence the crystalline structure^8^. The XRD pattern of MHCO/GPTMS-ZnO composites (Fig. 5c) contains the peaks of ZnO, whereas the peak intensities are relatively lower in comparison with those of pure ZnO. The results indicate that ZnO exists in MHCO/GPTMS-ZnO composites and the crystallinity ZnO decreases because of low content of ZnO in MHCO/GPTMS- ZnO composites.

**Figure 6:**
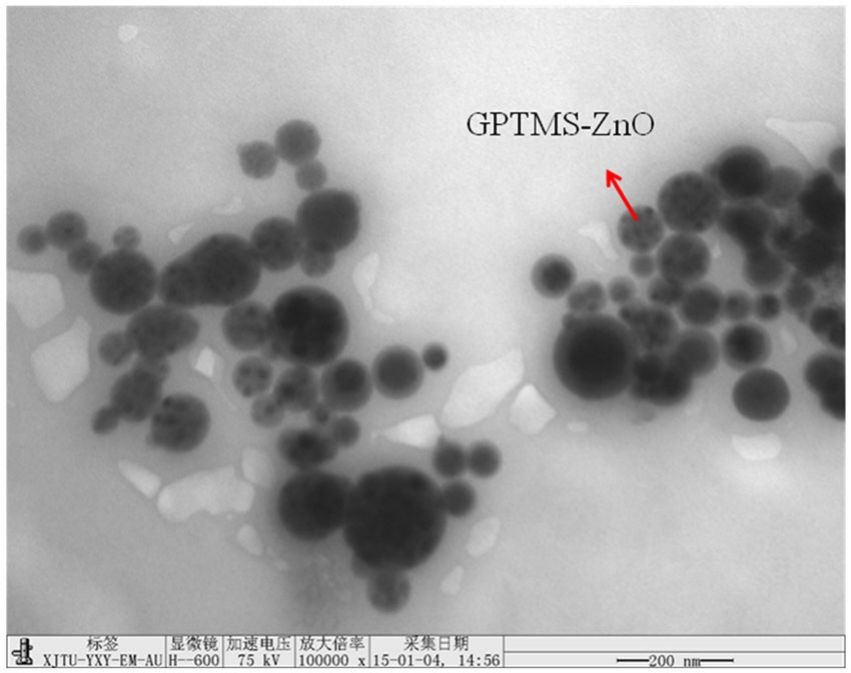
TEM image of MHCO/GPTMS-ZnO composites.

**Figure 7:**
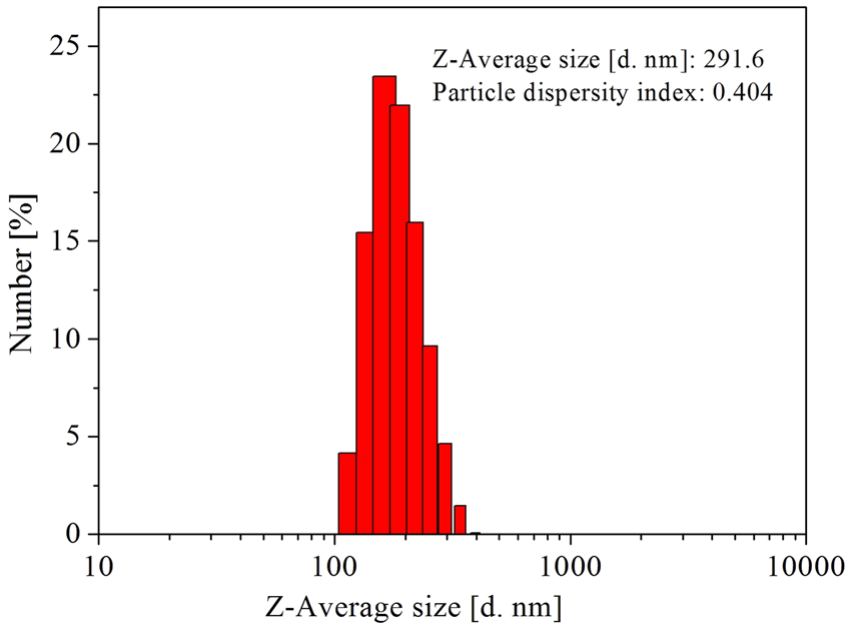
DLS of MHCO/GPTMS-ZnO composites.

Particle size distribution of fatliquoring agent emulsion is an important index of fatliquoring agent, which has an influence on fatliquoring agent penetration into leather structure^16^. The morphology and structure of MHCO/GPTMS-ZnO composites emulsion were observed by the TEM and DLS results as shown in Fig. 6 and Fig. 7. TEM in Fig. 6 shows that MHCO/GPTMS-ZnO composites emulsion is composed of spherical particles with diameters of 50~300 nm and GPTMS-ZnO nanoparticles are impregnated in MHCO. This is because MHCO having amphiphilic groups plays a role of the surfactant, which can wrap the hydrophobic GTPMS-ZnO nanoparticles and unreacted HCO, forming the spherical latex particles. The results from DLS in Fig. 7 reveal that MHCO/GPTMS-ZnO composites have narrow size distribution, uniform morphology, and an average diameter of 291.6 nm, which is consistent with those observed from TEM image. On the basis of all of the above FT-IR spectra, XRD patterns, DLS and TEM observations, it can be seen that MHCO/GPTMS-ZnO composites have been obtained by the present method.

### Properties of MHCO/GPTMS-ZnO composites

**Figure 8:**
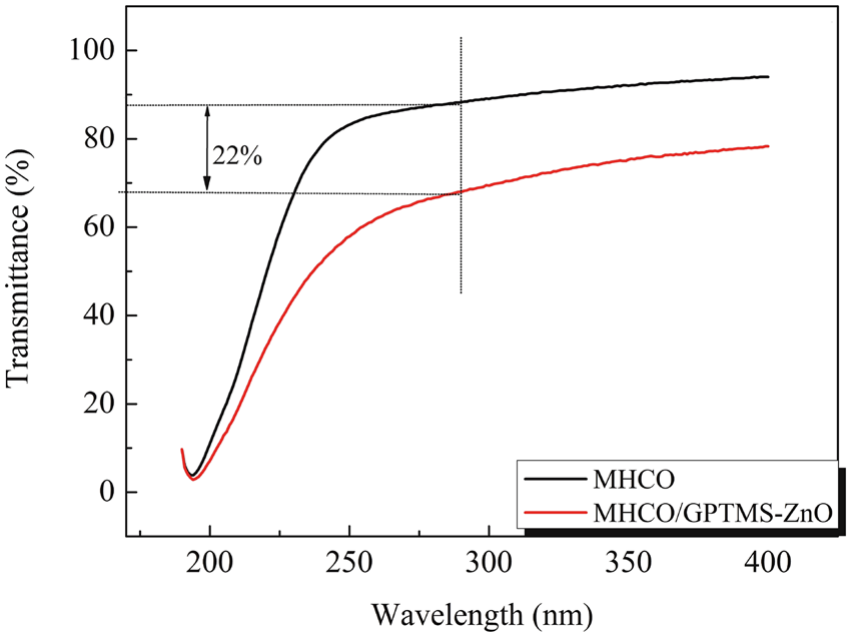
UV transmittance spectra of MHCO/GPTMS-ZnO composites and MHCO.

MHCO/GPTMS-ZnO composites and MHCO were characterized by UV spectrometer to investigate the effect of GPTMS-ZnO on anti-UV performance of emulsion. Figure 8 illustrates the UV transmittance spectra of MHCO/GPTMS-ZnO composites and MHCO. The transmittance of MHCO/GPTMS-ZnO composites is lower than that of MHCO. When the wavelength of UV light is 290 nm, the transmittance is decreased by 22%. It is attributed to the enhanced anti-UV performance for the presence of high UV absorption ZnO in MHCO/GPTMS-ZnO composites.

The stability of MHCO/GPTMS-ZnO composites is determined by observation and whether phase separation will occur on standing for six months^17, 18^. The observation results show that MHCO/GPTMS-ZnO composites are stable without any phase separation as oil and water for more than 6 months.

Combined DLOV colloid stability theory with zeta potential to further research the stability of the composites, colloid particles attract each other because of van der Waals force and repulsion when approaching each other due to overlapping of the electric double layers. Finally the stability of the colloid depends on the relative value of the two effects mentioned above. Zeta potential is a measure of the mutual repulsion between particles or attractive strength, and its significance lies in zeta potential value associating with the colloid dispersion stability. The higher the Zeta potential is, the more stable the system will be. The test shows that zeta potential of the composites is −32 mV. It indacates that the MHCO/GPTMS-ZnO composites are stable.

### Properties of fatliquored leathers

MHCO/GPTMS-ZnO composites, MHCO and commercial product were applied to leather processing in order to clearly understand the effect of GPTMS-ZnO on properties of leathers including yellowing resistance, softness and strength property. Table 1 shows the properties of fatliquored leathers.

**Table 1:** Properties of leathers fatliquored with different fatliquoring materials.

Experiment number	Experiment I	Experiment	Experiment III
Fatliquoring materials	MHCO/GPTMS-ZnO	II MHCO	Commercial
Dosage of fatliquoring materials	15 wt%	15 wt%	product 15 wt%
Anti-yellowing rating [class]	4–5	3–4	3
Average softness, [mm]	7.73	6.52	6.84
Average tensile strength, [MPa]	17.63	15.23	16.86
Average tear strength, [N/mm]	25.08	19.57	20.48
Average elongation at break, [%]	77	63.22	66.27

Experiment I-15 wt% MHCO/GPTMS-ZnO, Experiment II-15 wt% MHCO, Experiment III-15 wt% commercial product.

**Figure 9:**
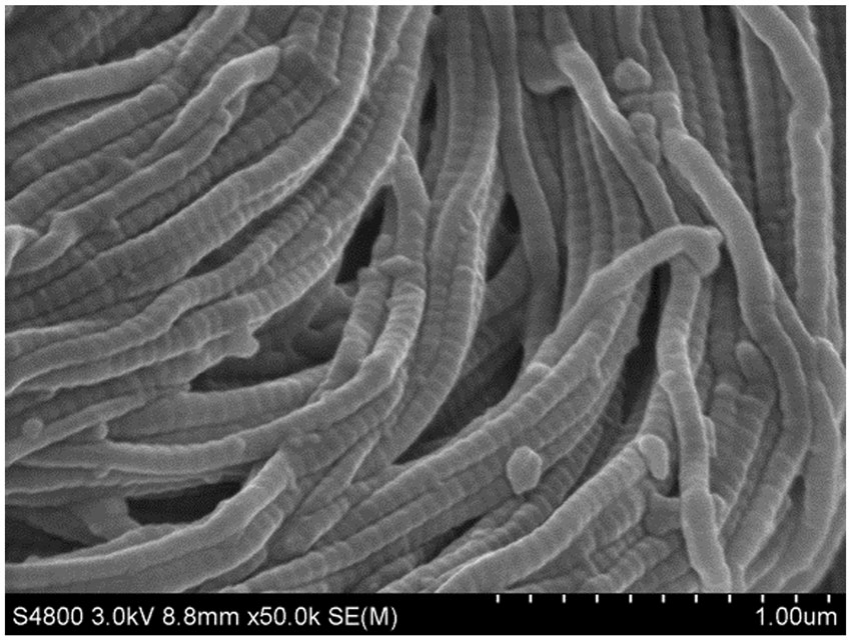
SEM image of cross-section of leather treated by MHCO/GPTMS-ZnO composites.

**Figure 10:**
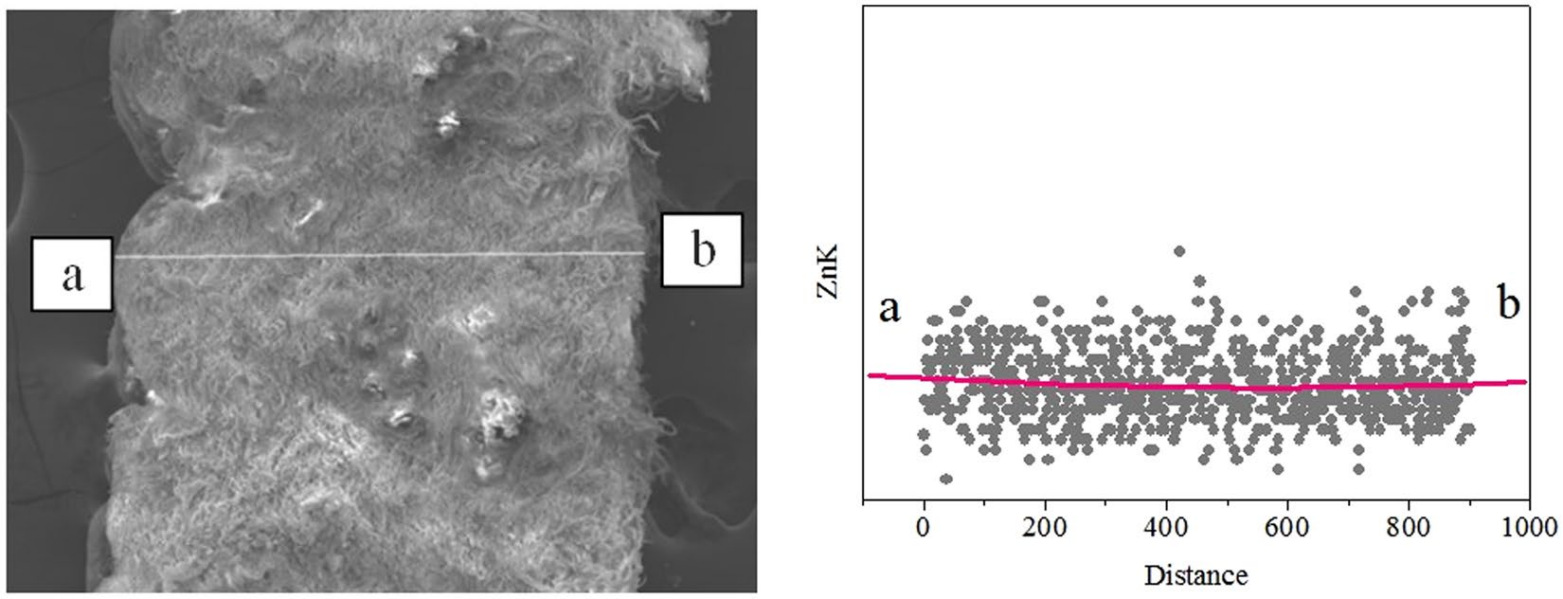
SEM-EDS of the longitudinal section of leather fatliquored by MHCO/GPTMS-ZnO composites.

**Figure 11:**
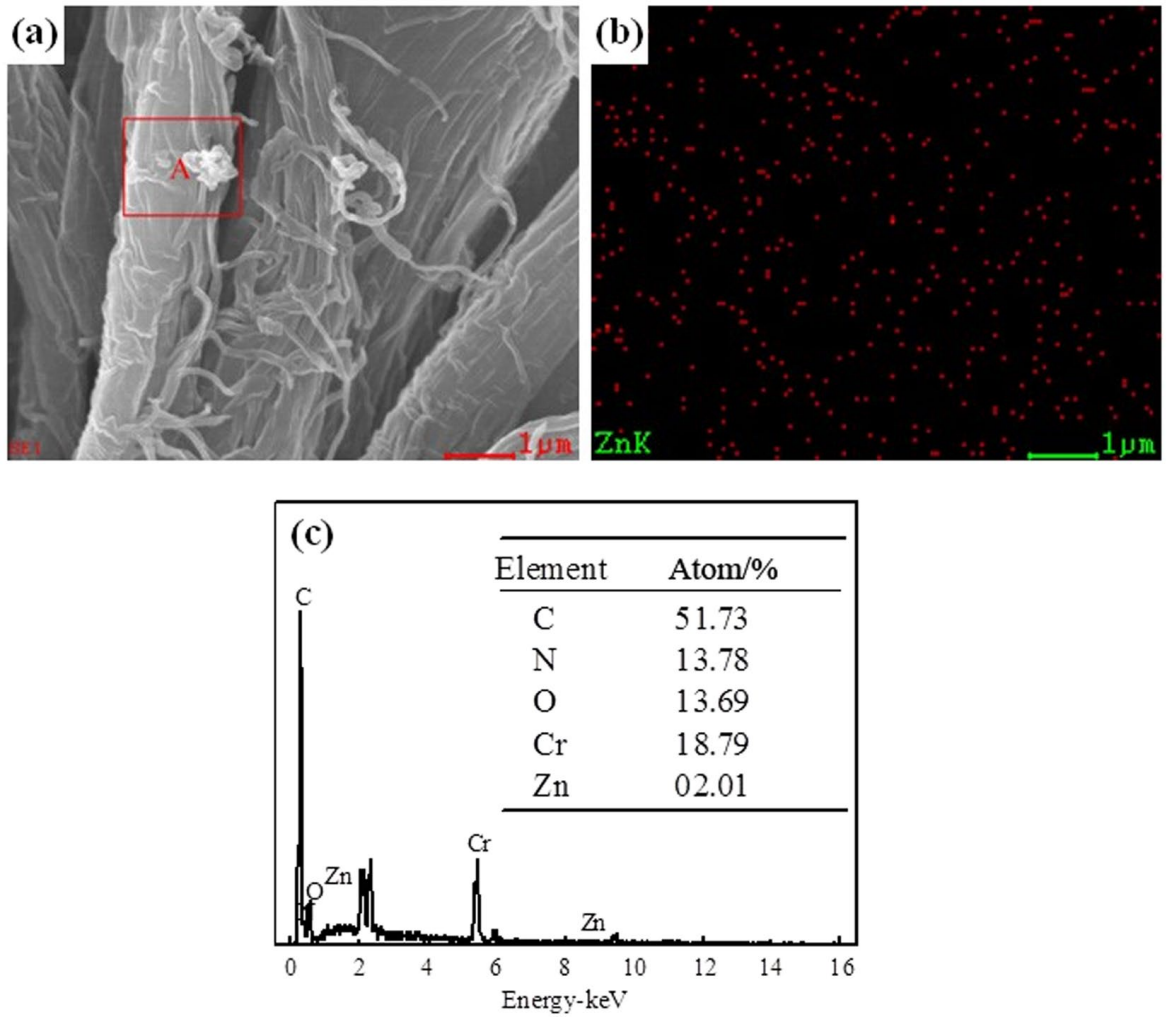
SEM-EDS of cross-section leather fatliquored by MHCO/GPTMS-ZnO composites.

From Table 1, it can be found that the anti-yellowing property of leather fatliquored by MHCO is higher than that of commercial product, and the anti-yellowing performance of leather treated with MHCO/GPTMS-ZnO composites is the best. The saturation of fatliquoring agent has a significant effect on the anti-yellowing performance of fatliquored leathers fibers. Fatliquoring agent with high saturation is more stable and less susceptible to oxidation under UV irradiation^19^. Based on FT-IR testing, it is known that MHCO/GPTMS-ZnO has a high saturation. It is more stable under UV irradiation and is not easily oxidized by oxygen in the air, thereby inhibiting the autoxidation reaction of oil and slowing the aging of leather fibers. Meanwhile, ZnO nanoparticles are uniformly distributed in the surface and the pores of the skin collagen fibers, which can absorb ultraviolet light and reduce the activity point of collagen fibers on ultraviolet light, thus reducing the damage of solar ultraviolet irradiation on the triple helical structure of leather collagen. Moreover, the interfacial interactions between ZnO nanoparticles and collagen fibers may enhance the ability of leather collagen fibers to resist ultraviolet light. This can be attested completely by the SEM image of cross-section of leather treated with MHCO/GPTMS-ZnO composites (Fig. 9, Fig. 10 and Fig. 11). Many ZnO nanoparticles can be seen clearly in the cross-section of fatliquored leather (as shown in particulate matter) in Fig. 9. Figure 10 shows Zn atom can evenly disperse from grain side to flesh side, which illustrates MHCO/GPTMS-ZnO composites can permeate from the outside to the interior of the leather collagen fibers. Figure 11 (b) and (c) show Zn atom can evenly dispersed among leather fibers and Zn element mass fraction is 2.01%. Thus, GPTMS-ZnO evenly disperses among leather fibers. The anti-yellowing performance of leather fatliquored with commercial product is worst, maybe because the commercial product contains a large number of unsaturated double bonds, easy to make the fatliquored leather yellow due to unsaturated double bonds fracture and autoxidation reaction of oil molecules^19^.

**Figure 12:**
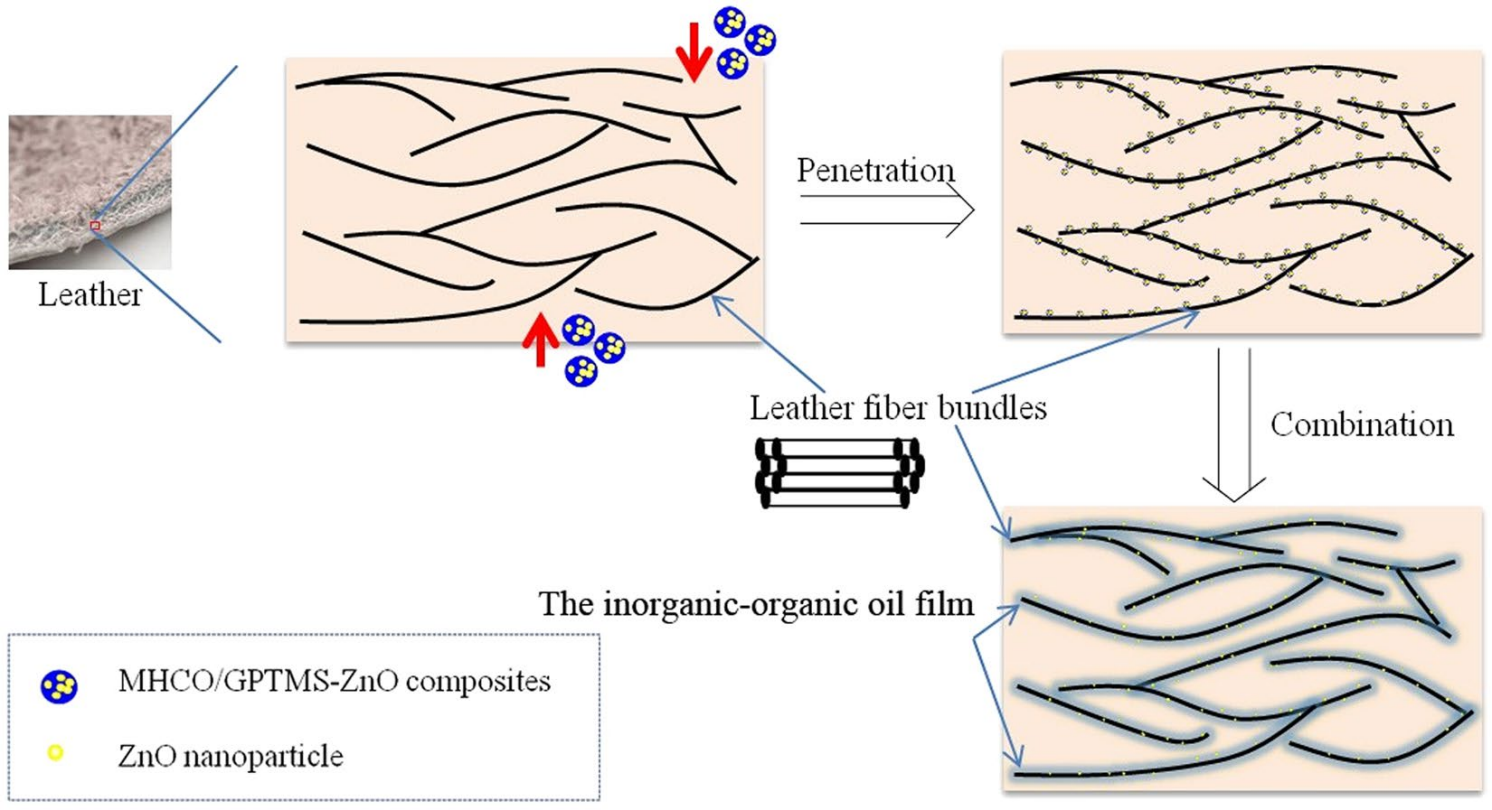
Schematic illustration of UV resistance of leather treated with MHCO/GPTMS-ZnO composites.

Based on above discussion and analysis of the anti-yellowing performance results, the possible scheme of anti-yellowing performance of leather fatliquored with MHCO/GPTMS-ZnO composites is shown in Fig. 12. MHCO/GPTMS-ZnO composites can permeate from the outside to the interior of the leather collagen fibers and wet the surface of leather collagen fibers because of hydrophilic groups in the composites. Moreover, by adjusting the pH of reaction medium, the composites can combine with the collagen fibers and form a continuous inorganic-organic oil film coating on the collagen fibers. The inorganic-organic oil film can lubricate the leather collagen fibers and prevent or decrease the damage of collagen structure under UV irradiation. Meanwhile, ZnO exposed to the outside of inorganic-organic oil film has a good absorbtionto ultraviolet light, weakening the aging effect of ultraviolet light on the collagen fibers.

Mechanical properties of fatliquored leathers are very important for leather including tensile strength, tear strength and elongation at break. It is found that the strength properties of leather fatliquored with MHCO/GPTMS-ZnO composites are better than those of MHCO. This is mainly attributed to the even distribution of GPTMS-ZnO in leather collagen fibers, which can further increase the distance among the molecular chains of leather collagen fibers and decrease the stress concentrations. The mechanical properties of leather fatliquored with MHCO are similar to those of leather fatliquored by commercial product.

Fatliquoring can lubricate and loosen the leather collagen fibers, increasing the softness of leathers. From Table 1, it can be found that softness of leather fatliquored with MHCO is considerable with that of the leather treated with commercial product. Compared with MHCO, the softness of leather fatliquored with MHCO/GPTMS-ZnO composites is higher. It is due to the fact that GPTMS-ZnO permeating into the leather collagen fibers, increasing the distance among the molecular chains and further obscuring the polar groups in the molecular chains and leading to the increasing of softness.

## Conclusions

In summary, MHCO/GPTMS-ZnO composites were successfully prepared by the reaction of succinic acid esterification-sulfonation process of hydrogenated castor oil. The introduction of GPTMS-ZnO could improve anti-UV performance of MHCO/GPTMS-ZnO composites. Leathers treated with MHCO/GPTMS-ZnO composites had outstanding anti-yellowing performance, mechanical properties and softness. MHCO/GPTMS-ZnO composites were composed of spherical particles with diameters of 50–300 nm and had narrow size distribution. ZnO was successfully modified by GPTMS and GPTMS-ZnO existed in MHCO/GPTMS-ZnO composites. MHCO/GPTMS-ZnO composites fabricated by this research and used in leather processing could solve the problem of leather fibers yellowing. The method and principle for preparing the present MHCO/GPTMS-ZnO composites can be extended to fabricate other inorganic-organic composites with different raw oil and inorganic nanoparticles.

## Methods

### Materials

Hydrogenated castor oil (HCO) was obtained from Guangzhou Jinggang Chemical Co., Ltd. Aqueous ammonia solution (25–28 wt%), sodium bisulfite (NaHSO_3_), maleic anhydride and 3-Glycidoxy propyl trimethoxy silane (GPTMS) were all purchased from Tianjin Kemiou Chemical Reagent Co., Ltd. and used as received. ZnO nanopowder with a diameter of 30 nm was purchased from Qinhuangdao Taijihuan Nano Products Co., Ltd.

### Surface modification of nano-ZnO

The surface of ZnO nanoparticles was activated by a silane coupling agent 3-Glycidoxy propyl trimethoxy silane (GPTMS). The ZnO nanoparticles (1.0 g) were added into a solution of ammonium hydroxide (1.4 mL), deionized water (20 mL) and absolute ethanol (60 mL), and then the mixed solution was sonicated for 1 h. Next, the suspension solution was charged into a 250 mL three-neck round-bottom flask, followed by addition of a mixture of GPTMS (0.04 g) and absolute ethanol (20 mL). The above mixture was refluxed at 60 °C under constant stirring for 2 h. The precipitate was centrifuged, washed with absolute ethanol three times to remove the residual silane, and then dried in a vacuum oven at 60 °C for 24 h to yield dried GPTMS-ZnO nanopowder for further use.

### Preparation of modified hydrogenated castor oil/GPTMS-ZnO (MHCO/GPTMS-ZnO) composites

MHCO/GPTMS-ZnO composites were prepared using the following procedure. Firstly, hydrogenated castor oil (20 g) and GPTMS-ZnO nanopowder (2.0 g) were charged into a 250 mL three-neck round-bottom flask, and then heated to 95 °C under constant stirring for about 30 min, followed by addition of maleic anhydride (7.2 g). The reaction was allowed to proceed for 3 h and then cooled to 65 °C, followed by addition of a solution containing 7.4 g of NaHSO_3_, 6 mL of aqueous ammonia solution and 40 mL of deionized water with vigorous stirring for 1 h. Subsequently, 7 mL of aqueous ammonia solution was added into the above mixture to modulate the reaction to pH of 7.0 under constant stirring for 2 h. At last, the final product was obtained after adjusting the solid content to 40% and stirring 30 min.

### Application

Fatliquoring process has been carried out to assess the applicability of prepared composites on leather. The wet blue of sheep was taken for fatliquoring. The leather was cut into 20 × 20 cm size samples. The weight of leather samples was recorded. The effect of GPTMS-ZnO in fatliquoring agent on anti-yellowing performance of treated leather has been studied. Fatliquoring experiments were carried out according to detailed process in Table 2. After fatliquoring, leather was dried in free state, and then it was mechanically softened by dry drumming.

**Table 2:** Wet-blue fatliquoring process.

Process	Amount [%]	Agent	Temperature [°C]	Time [min]	pH	Comments
Wetting back	200	Water	45			
	0.50	Formic acid		30	3.5	
	0.30	Wetting agent				Drain
Washing	300	Water	45	15		Drain
Retanage	100	Water	40	240		
	5	Chromium salt			4.0–4.2	Leave overnight, next morning run for 30 min, drain
Neutralization	200	Water	35			
	2	Sodium formate		30		
	1.0–1.5	Sodium bicarbonate		2 × 15 + 60	5.5–5.8	Drain
Washing	200	Water	35	15		Drain
Fatliquoring	150	Water	55			
	15	Fatliquoring agent^a^		90		
	2	Formic acid		3 × 20 + 30	3.8–4.0	Rinse, horse up

^a^ Experiment I-15% MHCO/GPTMS-ZnO, Experiment II-15% MHCO, Experiment III-15% commercial product.

### Characterization

A Bruker VECTOR-22 FT-IR spectrometer was applied to gain the FTIR spectra of bare ZnO nanoparticles, modified ZnO nanoparticles, MHCO and MHCO/GPTMS-ZnO composites. SEM-EDS of MHCO/GPTMS-ZnO composites are characterized by FEI ESEM. The overall crystalline phase of bare ZnO, GPTMS-ZnO and MHCO/GPTMS-ZnO composites were determined by XRD measurement on a X-ray diffractrometry (Rigaku, D/max-2200, Japan). XRD sample was prepared by flattening the sample in the sample container using a glass slide. Radial scans of intensity versus scattering angle (2*θ*) were recorded from 20 to 70° using a Cu Kα radiation. Transmission electron microscope (Hitachi H-600, Japan) was used to observe the morphologies of MHCO/GPTMS-ZnO composites. The samples of MHCO/GPTMS-ZnO composites for TEM observation were diluted with deionized water and treated for 5 min under ultrasound and then dried onto carbon-coated copper grids before examination. The particle size and size distribution of MHCO/GPTMS-ZnO composites were determined by Zeta PALS dynamic light scattering detector (Nano-ZS, Malvern Instruments Ltd., UK) at 25 °C. The MHCO/GPTMS-ZnO composites sample for DLS characterization was diluted with deionized water and treated for 5 min under ultrasound. UV-vis absorption and transmittance of MHCO/GPTMS-ZnO composites and MHCO were measured for estimating anti-UV ability. MHCO/GPTMS-ZnO composites and MHCO were diluted with deionized water to a concentration of 0.002% by mass. The absorption and transmittance of MHCO/GPTMS-ZnO composites and MHCO were measured using UV-vis spectrometer (TU-1900, China). The stability of MHCO/GPTMS-ZnO composites has been studied by observing phase separation (as oil and water). Field emission scanning electron microscope with energy-dispersive X-ray spectroscope (Hitachi S-48000, Japan) was used to investigate the morphological features of fatliquored leather samples. The cross-section and longitudinal section of fatliquored leather were sputter-coated with platinum, and then the morphological features of fatliquored leather samples were observed by SEM. Measurement of anti-yellowing performance of fatliquored leathers (HG/T 3689–2001, 2002) was performed with a GT 7035-NUA yellowing resistance test instrument (Guangzhou Dongguan Gotech Testing Machines Co., Ltd., China). The leather samples were taken following the standard procedure for sampling, air conditioning and thickness testing as published (ISO 2418: 2002, MOD; ISO 2419: 2002, MOD; ISO 2589: 2002, MOD, 2005). Mechanical properties of fatliquored leather such as tensile strength, elongation at break (ISO 3376: 2002, MOD, 2005) and tear strength (ISO 3377–1: 2002, MOD, 2011) were tested on a GT-U55 multi-function material testing machine (Guangzhou Dongguan Gotech Testing Machines Co., Ltd., China). All the tests were carried out in the direction of both parallel as well as perpendicular to backbone (every direction was tested for three times) and then the average values were taken. Measurement of leather softness was carried out on GT-303 softness test instrument (Guangzhou Dongguan Gotech Testing Machines Co., Ltd., China). The measurement was realized with the use of the ring of 25 mm in diameter. In softness testing, each sample was determined in three different position and average values were taken.
